# End of Apnea Event Prediction Leveraging EEG Signals and Interpretable Machine Learning

**DOI:** 10.3390/bios15110732

**Published:** 2025-11-02

**Authors:** Hisham ElMoaqet, Abdullah Ahmed, Mutaz Ryalat, Natheer Almtireen, Matthew Salanitro, Martin Glos, Thomas Penzel

**Affiliations:** 1 Mechatronics Engineering Department, German Jordanian University, Amman 11180, Jordan; a.ahmed1@gju.edu.jo (A.A.); mutaz.ryalat@gju.edu.jo (M.R.); natheer.almtireen@gju.edu.jo (N.A.); 2 Center of Sleep Medicine, Charité-Universitätsmedizin Berlin, 10117 Berlin, Germany; matthew.salanitro@charite.de (M.S.); martin.glos@charite.de (M.G.); thomas.penzel@charite.de (T.P.)

**Keywords:** sleep apnea, EEG, machine learning

## Abstract

Obstructive sleep apnea is a prevalent sleep disorder with serious health implications. While previous studies focused on detecting apnea events, little is known about the factors that determine whether an apnea episode continues or terminates. Understanding these mechanisms is crucial for optimizing treatment strategies. In this study, we analyzed 30-s brain activity segments during continuous and ending apnea events to identify neurophysiological markers of event termination, with particular emphasis on the most influential EEG features. Frequency-domain and complexity features were extracted, and several ensemble machine learning models were trained and evaluated. Our results show that the Extra Trees model achieved the highest performance, with an accuracy of 0.88, F1-score for ending apnea of 0.87, and an area under the receiver operating characteristic curve of 0.95. Feature importance analyses and SHAP visualizations highlighted frequency-band energy, Teager–Kaiser energy, and signal complexity as key contributors. Temporal analyses revealed how these features evolve during apnea termination. These findings suggest that cortical activation and transient arousal processes play a decisive role in ending apnea events and may facilitate the development of more advanced adaptive or closed-loop sleep apnea therapies.

## 1. Introduction

Benjafield et al. [1] reported that many people around the world suffer from Obstructive Sleep Apnea (OSA). It is estimated that 936 million adults worldwide have mild to severe OSA. Floras [2] also highlighted that about 15% of the general population suffer from OSA. Sleep apnea is defined as repeated episodes of obstructive apnea and hypopnea during sleep, accompanied by daytime sleepiness or altered cardiopulmonary function, as explained by Strollo and Rogers [3] and Slowik et al. [4]. Qureshi et al. [5], Lévy et al. [6], and Abbasi et al. [7] highlighted that OSA affects almost every system in the body, including the cardiovascular, pulmonary, and neurocognitive systems, increasing the risk of hypertension, cardiovascular disease, stroke, pulmonary hypertension, cardiac arrhythmias, and altered immune function. In addition, OSA raises the likelihood of accidents due to sleepiness and somnolence. Ho and Brass [8] and Kandasamy and Almeeleebia [9] reported that obesity, particularly a high BMI and large neck circumference, is among the strongest risk factors for OSA, with prevalence increasing in older adults and men being more affected than women because of body-fat distribution and hormonal differences.

Solutions have been developed to treat obstructive sleep apnea, one of which is positive airway pressure (PAP) machines. Kakkar et al. [10] and Weiss et al. [11] describe PAP therapy as a standard treatment for patients with moderate-to-severe sleep apnea and report that it reduces the risk of cardiovascular events in treated patients compared to untreated patients. Pengo et al. [12] highlighted the impact of CPAP therapy on reducing blood pressure. Lv et al. [13] reported that CPAP significantly reduced systolic and diastolic blood pressure, total cholesterol, triglycerides, and fasting blood glucose levels in patients with OSA, although no significant effects were observed on lipoprotein cholesterol, waist circumference, or body mass index. Khattak et al. [14] also noted that CPAP improves breathing in OSA patients and can benefit patients with heart failure and reduced ejection fraction by reducing oxygen desaturation, systolic blood pressure, heart rate, and intrathoracic pressure during sleep. Durtette et al. [15] found evidence for short-term improvements in cognitive flexibility following CPAP treatment, as measured by the Trail Making Test Part B, while effects on other cognitive domains remain less clear.

There are also other types of PAP machines, such as auto-adjusting positive airway pressure (APAP) and bilevel positive airway pressure (BiPAP or BPAP). Xu et al. [16] showed that APAP dynamically adjusts pressure throughout the night based on real-time detection of airflow limitations and upper-airway resistance, thereby optimizing airway patency with the lowest effective pressure. Pinto and Sharma [17] described BiPAP as providing two distinct pressure levels—higher inspiratory positive airway pressure during inhalation and lower expiratory pressure during exhalation—offering additional ventilatory support compared to CPAP for patients who require assistance with both oxygenation and ventilation.

Yang et al. [18] discussed that CPAP remains the primary treatment for obstructive sleep apnea in adults and children, and Weingarten [19] highlighted that CPAP is recognized as a cost-effective option from payer and societal perspectives when compared to alternatives such as oral appliances, surgery, or hypoglossal nerve stimulation.

Despite the usefulness of PAP machines, they also have downsides, including nasal dryness, dry mouth and throat, middle-ear pressure, ear pain, and aerophagia (swallowing air) [20]. Castrogiovanni and Bonsignore [21] have raised concerns about potential adverse cardiovascular effects associated with CPAP in some contexts, reporting signals such as elevated angiopoietin-2 and suggesting that higher therapeutic pressures may contribute to endothelial inflammation in susceptible patients. These observations underscore the need for careful titration of CPAP settings and further investigation into long-term outcomes.

Randerath et al. [22] and Luong et al. [23] argue that, given the heterogeneity of OSA symptoms and outcomes, a personalized approach beyond AHI-based severity classification is needed. In line with this, Patil et al. [24] concluded that evidence is currently insufficient to recommend PAP solely for improving cardiovascular outcomes, which motivates the search for more sophisticated, individualized treatment strategies. Data-driven or machine learning approaches could play a key role in defining patient-specific phenotypes by integrating polysomnographic, clinical, and outcome data to enable more precise and individualized therapies.

Given some of the downsides associated with CPAP machines, innovative solutions are needed to maximize patient comfort and recovery. This paper proposes a novel method of detecting when an apnea event ends in a patient. This information could be used to apply air pressure only when needed, thereby increasing the effectiveness of PAP therapy and potentially improving adherence.

To support this direction, it is important to consider the clinical and physiological significance of apnea termination. While substantial efforts have been directed toward detecting the presence and onset of OSA events, comparatively little is known about the mechanisms and clinical consequences of apnea termination. The end of an apnea event often coincides with abrupt autonomic activations, EEG arousals, and blood-pressure surges, all of which contribute to sleep fragmentation and cardiovascular strain. Predicting the termination phase could not only shed light on the neurophysiological mechanisms underlying event resolution but also facilitate the development of adaptive therapeutic interventions.

## 2. Materials and Methods

**Figure 1:**
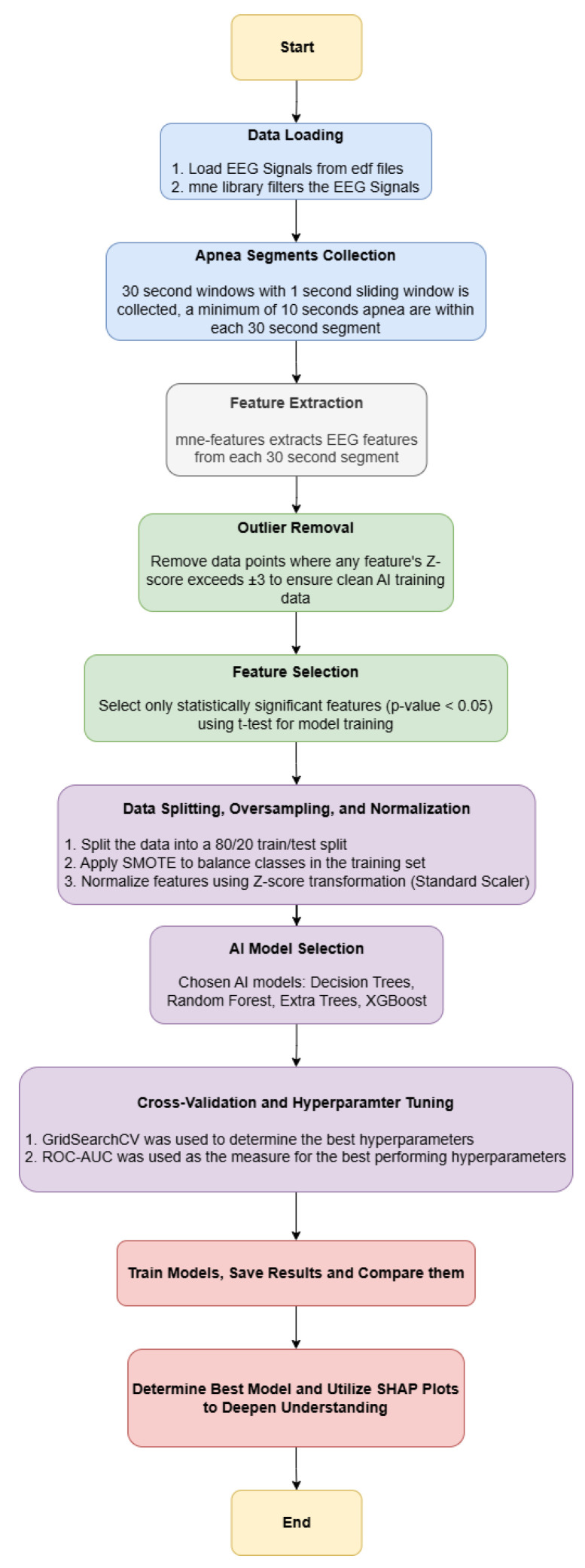
Flowchart Demonstrating the Methodology of the Paper.

This section delves into the details of our methodology, data collection, cleaning and preprocessing, feature extraction and selection, outlier removal, the machine learning models that were employed. The flowchart in Figure 1 highlights the approach of this paper.

### 2.1. Data

The data used originated of *n* = 68 sleep recordings performed at Charité-Universitätsmedizin Berlin of patients suffering from OSA as part of the European Sleep Apnea Database (ESADA). Patients gave their written consent for participation and the study was reviewed by the local ethics committee of the Charité-Universitätsmedizin Berlin, Germany with approval no. EA/1/139/7 according to the “Declaration of Helsinki” rules. Only the EEG signals (C4-A1 in particular) of sleep recordings were used for the purposes of analyzing the patients’ sleep and inputting them into the machine learning models for end of apnea prediction. The method behind the collection of data focused only the parts that contained apnea events within them from the sleep recordings. Obstructive, central and mixed apnea events were included in the dataset. A 30 s segment was chosen to do the analysis and each 30 s segment is a sample. The predefined segment also had a sliding window of 1 s. Each 30 s window must have at least 10 s of apnea within it. The reason for this is to increase the number of data samples and to keep track of the changes within the time frame of that apnea event. A 30-s segment containing at least 10 s of apnea was labeled as ending apnea if the apnea terminated within the segment; otherwise, it was labeled as continuous apnea. In total, 5004 apnea episodes were identified and used for training and evaluation in this study.

The experiments were conducted using Python version 3.12.3. The computations were performed on a system equipped with an AMD Ryzen 7 8845HS processor (3.80 GHz), 32 GB RAM, and an NVIDIA RTX 4070 Laptop GPU.

The EEG signal was filtered using the mne library (version: 1.9.0) [25]. It applied an IIR filter to retain frequencies between 0.1 Hz and 32 Hz. This effectively removed unwanted low-frequency drift and high-frequency noise. It also preserved relevant neural activity within this range. This made the EEG signals ready for the feature extraction process.

### 2.2. Feature Extraction from EEG Signals

To analyze EEG signals effectively, we extracted various features using the mne_features library (version: 0.3), which provides a standardized framework for computing diverse signal characteristics [25]. The features were computed from 30-s EEG segments and describe different aspects of the signal. These extracted features are grouped into the following categories.

#### 2.2.1. Time-Domain Features

Time-domain features describe **signal amplitude variations and statistical properties** over time. These features help in analyzing waveform morphology and identifying patterns in neural activity.

**Mean**: Represents the average amplitude of the EEG signal in a given time segment. It gives a basic measure of signal intensity.

**Standard Deviation**: Indicates the variability of the signal around its mean value. A higher standard deviation suggests greater fluctuation in amplitude.

**Root Mean Square (RMS)**: Measures the overall signal power, considering both magnitude and duration of fluctuations.

**Variance**: Represents the degree of dispersion in the signal. A higher variance suggests a less stable waveform.

**Kurtosis**: Describes how much of the signal’s energy is concentrated in extreme values. High kurtosis indicates sharp peaks, while low kurtosis suggests a flatter distribution.

**Skewness**: Indicates asymmetry in the signal distribution. A positive skew suggests that higher amplitude values are more common, whereas a negative skew indicates more frequent lower amplitude values.

**Line Length**: Measures the total variation in the EEG waveform, reflecting signal complexity and the presence of rapid amplitude changes.

**Decorrelation Time**: Describes how long it takes for the signal to lose its correlation with itself over time. A shorter decorrelation time suggests rapid changes in the signal pattern.

#### 2.2.2. Frequency-Domain Features

Frequency-domain features describe how signal energy is distributed across different frequency ranges. This helps in understanding oscillatory activity and identifying dominant rhythms in the EEG.

**Power Spectral Density (PSD)**: Quantifies how the signal’s power is distributed across different frequency bands, helping to identify dominant frequency components.

**Energy in Frequency Bands**: Measures the energy content in specific frequency ranges, commonly divided into:

- **band0 Delta (0.5–4 Hz):** Slow oscillations often linked to deep relaxation and low activity states.
- **band1 Theta (4–8 Hz):** Mid-range oscillations associated with transitions between different cognitive states.
- **band2 Alpha (8–12 Hz):** Rhythms often related to relaxed but alert states.
- **band3 Beta (12–30 Hz):** Higher-frequency activity typically linked to active cognitive processes and alertness.

The balance of power across these bands can reveal different brain states and functional characteristics.

#### 2.2.3. Entropy-Based Features

Entropy features assess the randomness, complexity, and predictability of a signal. These are useful for determining how structured or irregular the EEG pattern is.

**Sample Entropy**: Estimates the complexity of the signal by evaluating how often patterns repeat over time. Higher values suggest more randomness.

**Spectral Entropy**: Measures the distribution of power across frequencies. A high spectral entropy value indicates that power is spread across multiple frequencies, while a lower value suggests a dominant frequency component.

**Approximate Entropy**: Similar to sample entropy but less sensitive to noise. It provides an estimate of the predictability of fluctuations in the signal.

#### 2.2.4. Nonlinear Energy Features

These features measure instantaneous energy fluctuations in the EEG, helping to identify abrupt changes in neural activity.

**Teager–Kaiser Energy (TKE)**: Captures sudden bursts of energy by analyzing small, local changes in the signal. It is particularly useful for detecting rapid variations in EEG dynamics.

#### 2.2.5. Fractal and Complexity Measures

These features assess the self-similarity and irregularity of EEG waveforms, providing insights into the complexity of neural activity.

**Higuchi’s Fractal Dimension**: Estimates the degree of signal roughness, with higher values indicating more complex, irregular patterns.

**Katz’s Fractal Dimension**: Measures the structural complexity of the EEG waveform; a more irregular waveform leads to a higher Katz fractal dimension.

**Hjorth Parameters**: Provide statistical descriptions of the signal’s mobility and complexity:

- **Hjorth Mobility:** Measures how much the signal’s frequency content varies over time.
- **Hjorth Complexity:** Assesses the rate of change in the signal’s frequency characteristics.

By integrating these features, machine learning models and statistical analyses can effectively characterize EEG patterns, facilitating applications in sleep medicine, particularly in detecting and predicting the resolution of apnea episodes. This enables more accurate monitoring, diagnosis, and intervention strategies for sleep-related breathing disorders.

### 2.3. Data Preprocessing

Before inputting the extracted features into the machine learning models, necessary preprocessing steps were carried out, as EEG signals naturally contain noise. Handling this noise is a vital step to prevent the machine learning models from being misled by irrelevant variations, which could degrade their performance.

First, outlier removal was performed using the Z-score method, which standardizes each feature by subtracting the mean and dividing by the standard deviation. The Z-score for each data point is computed as:

(1) $$Z=\frac{x-\mu }{\sigma }$$

where *x* is the feature value, μ is the mean, and σ is the standard deviation of that feature. Any data points with a Z-score greater than 3 or less than −3 were considered extreme outliers and removed, as they could distort the learning process of the machine learning models. This step ensures that abnormal values, possibly caused by artifacts or sensor malfunctions, do not negatively impact training.

Next, feature selection was applied to retain only the most statistically significant features. A two-sample *t*-test on the means was conducted between the two classes (continuous vs. ending apnea), assessing whether each feature exhibited a significant difference in mean values. Only features with a *p*-value less than 0.05 were considered statistically significant and retained for training. This step removes redundant or irrelevant features, improving model generalization and reducing computational complexity. 36 features were used after the feature selection process.

After feature selection, the data was split into a 80/20 train-test split, and both sets were normalized independently using StandardScaler (Z-score normalization), which transforms features to have zero mean and unit variance. Finally, SMOTE (Synthetic Minority Over-sampling Technique) was applied to address class imbalance by generating synthetic examples for the minority class, ensuring the model is not biased toward the majority class [26]. After applying SMOTE, the number of samples in the minority class is increased to match that of the majority class.

### 2.4. Machine Learning Models

A multitude of machine learning models were tested to determine the one whose performance was most effective in the binary classification task of classifying whether a 30 s segment was either a continuous or an ending apnea event. The machine learning models that were experimented on where, Decision Trees, Random Forest, XGBoost, Extra Trees.

#### 2.4.1. Decision Trees

A decision tree is a hierarchical model that follows a tree-like structure, consisting of a root node, branches, internal nodes, and leaf nodes. The root node serves as the starting point and represents the primary feature used for the first decision. Internal nodes correspond to feature-based tests, with each branch representing a possible outcome of the test. The process continues until reaching a leaf node, which contains the final classification or numerical prediction.

Decision trees are intuitive and closely resemble human decision-making processes, making them highly interpretable. By recursively partitioning the data and applying decision rules at each node, the model progressively refines its predictions, ultimately yielding a single outcome at each leaf. This structured approach allows decision trees to efficiently handle both classification and regression tasks [27,28].

#### 2.4.2. Random Forest

Random Forest (RF) is an ensemble learning algorithm based on the bagging (Bootstrap Aggregating) method, known for its robust performance in noisy datasets and weakly discriminative features. Unlike many other machine learning models, RF is insensitive to parameter initialization and provides high stability in classification tasks. The algorithm constructs multiple decision trees by randomly selecting subsets of training data through bootstrap sampling, ensuring that each tree is trained on a slightly different dataset. These trees operate independently, and the final classification is determined through a majority voting process, while regression tasks use an averaging approach to generate predictions.

RF is also widely utilized for feature selection due to its ability to handle high-dimensional and highly correlated variables. The permutation-based feature importance method evaluates a feature’s contribution by randomly shuffling its values and assessing the impact on model performance. Additionally, the Gini impurity metric is used to determine which features contribute most to minimizing classification uncertainty. To further refine feature selection, random forest cross-validation (rfcv) systematically reduces the number of predictors through a nested cross-validation process, ranking features based on their importance [29,30].

#### 2.4.3. XGBoost

XGBoost is a powerful ensemble learning method that employs boosting, a technique that combines multiple weak learners to form a strong predictive model. The algorithm constructs decision trees sequentially, where each tree aims to correct the misclassifications of the previous one. Initially, all input variables are assigned weights, and misclassified instances have their weights increased before being passed to the next tree.

The process begins with a base model that predicts the average of all possible classes for each data sample. The algorithm then calculates the residuals—the difference between actual and predicted values—and builds a new decision tree to model these residuals. Each decision tree in XGBoost performs binary splits on features to optimize prediction accuracy. For each split, the algorithm calculates information gain, which helps determine the best feature for partitioning the data. The information gain is derived using the similarity measure of different branches of the tree.

This iterative refinement continues as each new tree focuses on minimizing the residual errors of the previous one. Once the ensemble is complete, the final prediction is determined by aggregating outputs from all trees. XGBoost employs a soft voting mechanism to compute final class probabilities. Unlike hard voting, where the majority class is chosen outright, soft voting averages the weighted probabilities from all trees, leading to a more nuanced final decision. This probability-based approach also helps assess the uncertainty of predictions, which plays a crucial role in the proposed query function [31,32].

#### 2.4.4. Extra Trees

The Extra Trees Classifier (ETC), short for Extremely Randomized Trees, is an ensemble machine learning algorithm that enhances predictive performance by aggregating multiple uncorrelated decision trees. This method improves accuracy and robustness by leveraging randomness in both feature selection and decision tree construction.

Unlike traditional ensemble techniques such as Random Forests, which rely on bootstrapped subsets of data, ETC builds each decision tree using the entire dataset. Additionally, it introduces a higher degree of randomness by selecting split points completely at random rather than based on criteria like information gain or Gini impurity. This additional randomization increases the diversity among the trees, helping to balance bias and variance while improving generalization across various predictive tasks [33,34].

### 2.5. SHAP: Explainability in Machine Learning Models

Understanding how machine learning models make decisions is crucial, especially in high-stakes applications like healthcare. SHAP (SHapley Additive Explanations) is a powerful framework for explaining machine learning models by assigning feature importance scores based on game theory principles. It provides a consistent and interpretable approach to analyzing model predictions.

#### Key Benefits of SHAP

- **Global & Local Interpretability:** SHAP explains individual predictions while also summarizing feature importance across the entire dataset.
- **Consistent & Fair Attribution:** Built on Shapley values from cooperative game theory, ensuring a fair distribution of feature contributions.
- **Model Agnostic & Specific Support:** Works with both black-box models (e.g., deep learning) and complex machine learning models (e.g., XGBoost, SVM). Additionally, SHAP offers optimized explainers for tree-based models.
- **Visual Explanations:** Provides various visualization tools, such as summary plots, heatmap plots, and waterfall plots, to illustrate model behavior.

## 3. Results

This section highlights the results that were collected from the experiments with the various machine learning models using the Scikit-learn python library. Table 1 displays the number of the training samples (before SMOTE oversampling) and the testing samples and their distribution for each class after the z-score outlier removal process. Table 2 illustrates the performance of the machine learning models, the metrics Precision, Recall and F1-Score were used to demonstrate each machine learning model’s performance on the ending apnea class, The metrics Accuracy and AUC-ROC were used to judge the machine learning models’ performance as a whole. Table 3 highlights the best hyperparameters that were chosen for each of the machine learning models.

It can be observed from Table 2 that Extra Trees was the top-performing model across all tests conducted, achieving an accuracy of 0.88 and an AUC-ROC of 0.9504. Additionally, it recorded the highest F1-score for the ending apnea class at 0.87, demonstrating its effectiveness in identifying apnea events.

The XGBoost model also demonstrated highly competitive performance, with an accuracy of 0.87 and an AUC-ROC of 0.9440, making it another effective model. Its F1-score for the ending apnea class was 0.87, further confirming its reliability.

Random Forest, while still a strong performer, showed a slight drop in effectiveness compared to the other two ensemble models. It achieved an accuracy of 0.85 and an AUC-ROC of 0.9247, indicating that although it remained a viable choice, it did not generalize as well as Extra Trees and XGBoost. Its F1-score for the ending apnea class was 0.84, showing slightly lower precision and recall.

As expected, Decision Trees delivered the poorest performance among the tree-based models. Since ensemble methods such as Extra Trees, XGBoost, and Random Forest are designed to improve upon the weaknesses of individual decision trees, the standalone Decision Tree model struggled with an accuracy of 0.73 and an AUC-ROC of 0.7758. Its F1-score for the ending apnea class was 0.72, highlighting its limited generalization ability and tendency to overfit.

**Figure 2:**
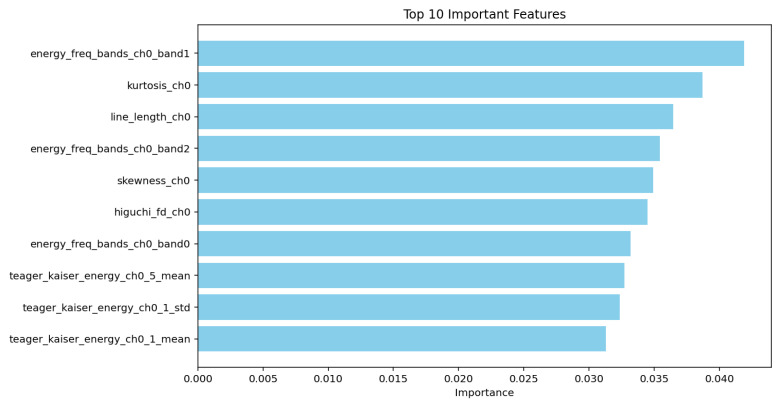
Figure illustrating Extra Trees Top Features.

Figure 2 depicts the top features that contributed the most to the Extra Trees model’s ability to classify the continuous and ending apnea classes effectively. The most influential feature, energy_freq_bands_ch0_band1 (4.1%), suggests that frequency band energy plays a crucial role in distinguishing apnea states, likely capturing respiratory signal variations. Other top features, such as kurtosis_ch0 3.7%) and skewness_ch0 (3.5%), indicate that higher-order statistical moments of the signal are key discriminators, highlighting differences in waveform distribution between apnea and non-apnea events. Additionally, line_length_ch0 (3.6%) and higuchi_fd_ch0 (3.5%) point to the significance of signal complexity and irregularity in apnea detection. The presence of teager_kaiser_energy_ch0_5_mean (3.3%), along with other Teager–Kaiser energy operator-based features, suggests that variations in energy dynamics also contribute to classification.

**Figure 3:**
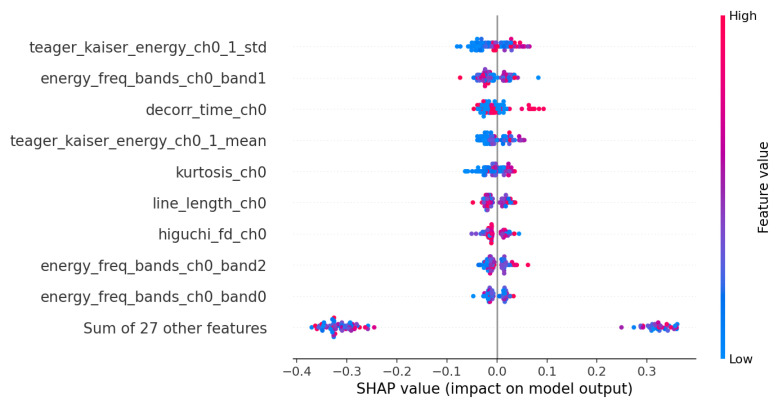
SHAP Beeswarm Plot.

**Figure 4:**
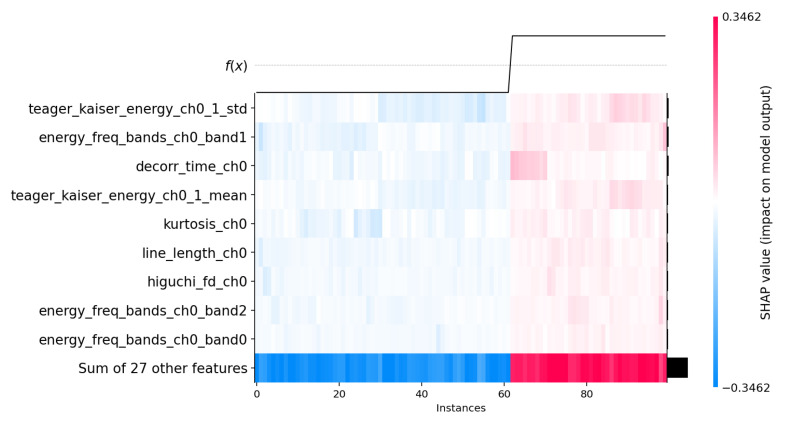
SHAP Heatmap Plot.

The SHAP beeswarm plot (Figure 3) provides complementary insights by illustrating how individual features influence specific classifications, with larger absolute SHAP values indicating stronger impact on model decisions. Notably, teager_kaiser_energy_ch0_1_std and teager_kaiser_energy_ch0_1_mean capture energy fluctuations in the respiratory signal, helping detect airflow pattern changes that distinguish apnea from normal breathing. Similarly, frequency-domain features such as energy_freq_bands_ch0_band1 and energy_freq_bands_ch0_band2 reflect variations in respiratory rhythms associated with apnea events. The SHAP heatmap (Figure 4) further reinforces these findings by visualizing the per-instance impact of each feature on model predictions, showing consistent contributions from these energy-based and statistical metrics across multiple instances. A unique feature highlighted by SHAP, decorr_time_ch0, may relate to signal stability or variations over time, potentially indicating irregular breathing patterns induced by apnea. Additionally, complexity-based features such as line_length_ch0 and higuchi_fd_ch0 continue to show strong contributions, emphasizing the importance of signal irregularity in apnea classification.

**Figure 5:**
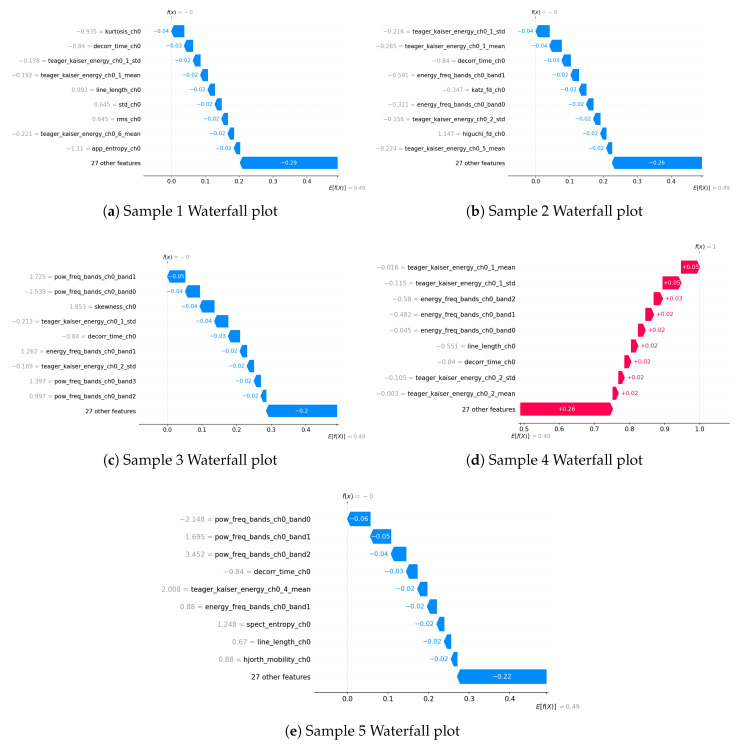
Comparison of waterfall plots across different samples.

The waterfall plots (Figure 5) provide a more granular, sample-specific perspective on feature importance, complementing the global rankings observed in the Extra Trees and SHAP analyses. Consistent with previous findings, **Teager–Kaiser energy features** and **frequency-domain energy bands** appear as dominant contributors across multiple samples, reinforcing their strong role in distinguishing apnea from normal breathing. Specifically, features such as teager_kaiser_energy_ch0_1_std, teager_kaiser_energy_ch0_1_mean, and energy_freq_bands_ch0_band1 are repeatedly observed, highlighting their predictive significance.

Moreover, higher-order statistical moments, such as kurtosis_ch0 and skewness_ch0, and signal complexity measures, such as line_length_ch0, occasionally emerge as influential factors. This suggests that signal distribution characteristics and irregularity contribute to classification in certain cases, even if they do not appear consistently across all samples.

Interestingly, power-related features, such as pow_freq_bands_ch0_band0 and pow_freq_bands_ch0_band1, emerge in some waterfall plots, despite not being highlighted in the global feature rankings of Extra Trees or SHAP. This indicates that while power features may not have strong predictive importance across the entire dataset, they can still play a notable role in specific cases, potentially capturing spectral characteristics of apnea that are more pronounced in certain instances.

**Figure 6:**
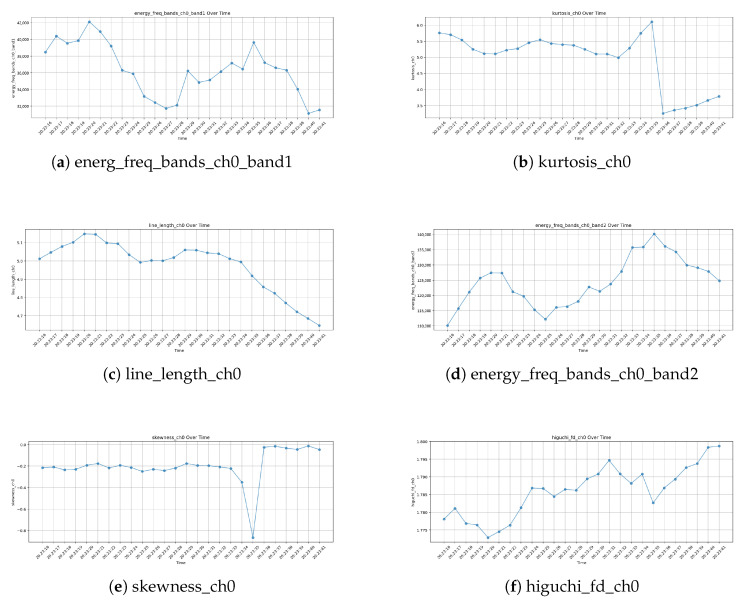
EEG Features Over Time Plots.

Figure 6 illustrates the temporal evolution of key EEG features, computed using a 30-s segmentation with a 1-s sliding window. These features offer critical insights into the neurophysiological fluctuations that occur at the termination of apnea events—a period marked by autonomic and cortical responses aimed at restoring normal breathing.

### 3.1. Energy in Frequency Band 1 (energy_freq_bands_ch0_band1)

This feature exhibits prominent fluctuations, characterized by distinct peaks and valleys, followed by a gradual decline. The reduction in spectral energy within this band toward the end of the event may correspond to a decrease in cortical arousal, as the brain transitions from a heightened activation state back to a more stable neural condition. This pattern suggests that spectral energy in this frequency range may serve as a marker of the post-apneic recovery process.

### 3.2. Kurtosis (kurtosis_ch0)

The kurtosis feature remains relatively stable for most of the segment but experiences a sharp increase just before apnea termination, followed by a rapid drop. This transient spike may be indicative of bursts of high-amplitude EEG activity, potentially corresponding to post-apneic arousals or abrupt shifts in brain state associated with the resumption of normal respiration. The subsequent drop suggests a return to baseline signal characteristics, reinforcing the hypothesis that transient cortical activations play a role in apnea resolution.

### 3.3. Line Length (line_length_ch0)

Initially, the line length feature increases, peaks around apnea termination, and then gradually declines. Given that line length is a measure of signal complexity, this pattern suggests that neural activity becomes more irregular leading up to apnea termination but stabilizes afterward. This could reflect the transient arousal response that often accompanies apnea resolution, followed by the recovery phase as homeostasis is restored.

### 3.4. Energy in Frequency Band 2 (energy_freq_bands_ch0_band2)

In contrast to Band 1, this feature follows an increasing trend with oscillatory variations, eventually exhibiting a slight decline post-apnea. The rise in spectral energy in this band may indicate enhanced cortical activity linked to autonomic responses at apnea termination, possibly reflecting increased sympathetic nervous system engagement. The subsequent decline may correspond to a stabilization phase, as respiratory and neural systems gradually return to equilibrium.

### 3.5. Skewness (skewness_ch0)

Skewness remains relatively stable throughout the segment but exhibits a sharp drop at one point, followed by a recovery to a slightly higher level than its initial range. This transient shift may indicate abrupt changes in EEG signal asymmetry, potentially linked to sudden neural adjustments associated with apnea cessation and airflow resumption. The observed rebound suggests that apnea termination is accompanied by a restructuring of neural activity.

### 3.6. Higuchi Fractal Dimension (higuchi_fd_ch0)

The fractal dimension gradually increases over time, with notable fluctuations, indicating rising EEG complexity. This trend suggests that cortical activation becomes more dynamic leading up to apnea termination, possibly due to interactions between arousal mechanisms, autonomic regulation, and respiratory control. The fluctuations in fractal dimension highlight the intricate neural processes involved in apnea termination, before ultimately stabilizing.

Figure 2 illustrates the top 10 features for the best performing model (Extra Trees). Figure 3 and Figure 4 demonstrate 2 SHAP plots that provide a general explanation of the feature’s effects on the predicted samples, Figure 5 depicts 5 waterfall plots for 5 random samples to gain a better understanding of each feature’s contribution in the predictions.

Figure 6 illustrates the variations in the top 6 EEG features from Extra Trees over time, analyzed using 30-s segments with a 1-s sliding window.

## 4. Discussion and Conclusions

The results of this study emphasize the advantages of ensemble learning, particularly in handling complex patterns within EEG data for apnea classification. Among the models tested, the Extra Trees classifier achieved the highest performance (AUC-ROC = 0.95, F1 = 0.87), establishing it as the most effective approach for distinguishing between continuing and ending apnea events. These findings suggest that ensemble-based approaches provide robust and scalable solutions for predicting apnea termination, especially when dealing with numerous features and substantial sample sizes.

Feature importance analyses revealed significant overlap between Extra Trees rankings and SHAP-based visualizations, reinforcing the robustness of key predictive features. Frequency-band energy, Teager–Kaiser energy, and signal complexity metrics consistently emerged as highly informative, while SHAP highlighted temporal properties, such as decorr_time_ch0, demonstrating that local, instance-level insights complement global feature rankings. Additionally, by plotting how EEG-derived features evolve over time, we gained valuable insights into the dynamic neural patterns surrounding apnea termination, further reinforcing the link between neural activity changes and event resolution.

The most influential features energy_freq_bands_ch0_band1 (4.1%), kurtosis_ch0 (3.7%), line_length_ch0 (3.6%), higuchi_fd_ch0 (3.5%), and skewness_ch0 (3.5%) show that both spectral energy modulations and higher-order statistical descriptors are critical for classification. The Teager–Kaiser energy features further reflect transient bursts linked to cortical arousal and event resolution. The narrow importance range (3.3–4.1%) suggests that apnea termination is characterized by a distributed pattern of EEG dynamics rather than dependence on any single feature.

The top-ranked features, including band1–band2 energy, Teager–Kaiser energy, kurtosis, line length, and fractal dimension, also provide physiologically meaningful insights. Increased band1 (theta) and band2 (alpha–beta) energy likely reflect cortical arousal and autonomic activation that facilitate airway reopening. Elevated Teager–Kaiser energy indicates brief neural bursts consistent with motor or cortical drive at event termination. Peaks in kurtosis and line length capture transient, high-amplitude EEG irregularities typical of micro-arousals, while changes in fractal dimension suggest short-term cortical instability preceding recovery. Together, these findings highlight that apnea termination involves coordinated cortical activation and arousal processes rather than passive event cessation.

From a practical standpoint, the ability to predict apnea termination could be integrated into adaptive therapeutic frameworks. Although many apnea events resolve spontaneously, identifying those likely to persist versus those that will naturally terminate enables a more individualized approach to treatment. For example, incorporating such prediction models into auto-adjusting PAP (APAP) systems could allow dynamic modulation of airway pressure, applying higher pressure only when an ongoing event is unlikely to end spontaneously. This strategy could minimize unnecessary pressure elevations, enhance comfort, and potentially improve adherence. Furthermore, real-time predictions could support closed-loop biofeedback systems that adjust ventilatory support or deliver mild stimuli (e.g., brief pressure pulses or auditory cues) to promote event resolution before significant oxygen desaturation occurs. These applications demonstrate how apnea termination prediction can move beyond descriptive analysis toward intelligent, patient-specific therapy adjustment.

The EEG feature variations observed in this study support the hypothesis that apnea termination is an active process involving arousal-related and autonomic mechanisms, rather than a passive cessation of events. The interplay between spectral energy shifts, transient high-amplitude activity, signal complexity, and cortical reorganization underscores the potential of EEG-derived markers for both mechanistic insight and clinical prediction.

From a neurophysiological perspective, the EEG changes observed here are consistent with known markers of cortical arousal and autonomic activation during apnea resolution. Increases in band1 and band2 energy may correspond to transient surges in theta and alpha–beta activity associated with cortical arousals and the restoration of airflow, as described by Taylor et al. [35] and Younes [36]. Peaks in kurtosis and line length reflect brief high-amplitude bursts and signal irregularity typical of micro-arousals, also noted by Taylor et al. [35]. Furthermore, fluctuations in fractal dimension indicate transient cortical instability and reorganization before normalization, consistent with findings reported by Penzel et al. [37]. These associations position the model’s predictive EEG features within a recognized physiological framework, linking spectral and complexity measures to the neural processes underlying apnea termination.

To our knowledge, no prior studies have specifically addressed apnea termination. While previous research focused on apnea detection or onset prediction, this study introduces a novel task: predicting continuation versus termination of ongoing apnea events. Our methodology achieved high performance leveraging Extra Trees (AUC-ROC = 0.95, F1 = 0.87), demonstrating that apnea termination prediction is both feasible and clinically relevant.

Despite these encouraging results, several avenues for future research remain. Stage-specific analyses could determine whether termination-related features differ across REM and non-REM sleep. Larger, more diverse cohorts would help validate model generalizability, and incorporating apnea onset could enable full-cycle prediction. Exploring additional physiological signals, refining feature extraction, and integrating models into real-time adaptive PAP devices could further enhance clinical applicability.

In conclusion, this study advances both methodological and clinical understanding of apnea termination. By elucidating EEG features linked to event resolution, leveraging interpretable ensemble models, and analyzing feature dynamics over time, it provides a foundation for more targeted, adaptive OSA therapies and paves the way for intelligent, patient-specific interventions.

## Figures and Tables

**Table 1:** Class Distribution for Training and Testing Data Segments.

Dataset	Continuous Apnea Count	Ending Apnea Count
Training Data Segments	63,738	57,912
Testing Data Segments	15,935	14,478

**Table 2:** Comparison of Machine Learning Models Based on Ending Apnea Performance Metrics.

Model	Precision	Recall	F1-Score	Accuracy	AUC-ROC
Decision Tree	0.73	0.71	0.72	0.73	0.78
Random Forest	0.85	0.82	0.84	0.85	0.92
XGBoost	0.87	**0.86**	**0.87**	0.87	0.94
**Extra Trees**	**0.88**	**0.86**	**0.87**	**0.88**	**0.95**

**Table 3:** Best Hyperparameters for Each Model.

Model	Best Hyperparameters
Decision Tree	{max_depth: None, max_features: None, min_samples_leaf: 4, min_samples_split: 10}
Random Forest	{n_estimators: 500, max_depth: None, min_samples_split: 2, min_samples_leaf: 1, max_features: None, bootstrap: True}
XGBoost	{colsample_bytree: 0.8, gamma: 0.1, learning_rate: 0.1, max_depth: 15, min_child_weight: 1, n_estimators: 500, scale_pos_weight: 1.0, subsample: 0.8}
Extra Trees	{bootstrap: False, max_depth: None, max_features: None, min_samples_leaf: 1, min_samples_split: 2, n_estimators: 500}

## Data Availability

The data that supports the findings of this study are available from Charité–Universitätsmedizin Berlin, in Berlin, Germany, but restrictions apply to the availability of these data. The data sets in this research were used under license for the current study and therefore are not publicly available. Data sets of this study can be made available upon approval of a research request submitted to the corresponding author of this study.
